# Supplementary material for: Analysis of PPARγ Signaling Activity in Psoriasis
Source: Int J Mol Sci. 2021 Aug 10;22(16):8603. doi: 10.3390/ijms22168603 (PMC8395241; doi:10.3390/ijms22168603)
Supplement: Supplementary file 1 [file ijms-22-08603-s001.zip › Supplemental materials_Analysis of PPARg signaling activity in psoriasis/Pathway models/Models images and html files/PPARG expressed in cells assotiated with psoriasis/225679.html]

PBMC


# CellType PBMC

|  |  |
| --- | --- |
| URN | urn:agi-ncimcelltype:C1321301 |
| Total Entities | 45 |
| Connectivity | 6254 |
| Name | PBMC |

---

|  |  |
| --- | --- |
| ChildConcepts | PBMC proliferation |
|  | PBMC migration |
|  | PBMC transendothelial migration |
|  | PBMC apoptosis |
|  | PBMC population |
|  | PBMC development |
|  | PBMC count |
|  | PBMC growth |
|  | PBMC chemotaxis |
|  | PBMC proliferative response |
|  | PBMC function |
|  | PBMC death |
|  | PBMC adhesion |
|  | PBMC differentiation |
|  | PBMC aggregation |
|  | PBMC phagocytosis |
|  | PBMC infiltration |
|  | PBMC cytotoxicity |
|  | PBMC mediated cytotoxicity |
|  | PBMC phenotype |
|  | PBMC homing |
|  | PBMC extravasation |
|  | PBMC viability |
|  | PBMC priming |
|  | PBMC survival |
|  | PBMC rolling |
|  | PBMC interaction |
|  | PBMC distribution |
|  | PBMC repertoire |
|  | PBMC formation |
|  | PBMC damage |
|  | PBMC size |
|  | PBMC motion |
|  | PBMC homeostasis |
|  | PBMC invasion |
|  | PBMC motility |
|  | PBMC senescence |
|  | PBMC structure |
|  | PBMC diapedesis |
|  | PBMC stress |
|  | PBMC sequestration |
|  | PBMC fate commitment |
|  | PBMC ratio |
|  | PBMC spreading |
|  | PBMC disruption |

---

|  |  |
| --- | --- |
| Pathway | PPARG expressed in cells assotiated with psoriasis |
|  | Neighbors of PBMC |

---

|  |  |
| --- | --- |
| MedScan ID | 10000173 |

---

|  |  |
| --- | --- |
| Alias | mononucleated blood cell |
|  | circulating mononuclear cells |
|  | peripheral blood mononuclear cell |
|  | peripheral blood mononuclear cells |
|  | PBMCs |
|  | mononuclear blood cells |
|  | mononuclear blood cell |
|  | PBMC |
|  | peripheral blood mononuclears |
|  | peripheral blood mononucleated cells |
|  | peripheral mononuclear cells |
|  | peripheral blood mononucleated cell |
|  | mononucleated blood cells |

---

|  |  |
| --- | --- |
| NCIm ID | C1321301 |

---
